# Gene Expression Profiling Suggests Downregulation of Wnt Pathway Signaling With Aging

**DOI:** 10.1093/geroni/igaa057.465

**Published:** 2020-12-16

**Authors:** Allison Kuipers, Cara Nestlerode, Victor Wheeler, Iva Miljkovic, Joseph Zmuda

**Affiliations:** 1 University of Pittsburgh, Pittsburgh, Pennsylvania, United States; 2 Tobago Health Studies Office, Upper Scarborough, Tobago, Trinidad and Tobago

## Abstract

The wingless (Wnt) pathway is involved in many age-related diseases and conditions, including cancer and cardiovascular disease. However, the impact of aging on Wnt pathway signaling remains largely unknown. Therefore, we surveyed peripheral blood expression of 43 Wnt pathway genes and tested for association with age in 369 Afro-Caribbean men recruited from the population of Tobago (mean age 64 years, range 51-89 years). Gene expression was measured in counts per sample, then normalized and background subtracted. All expression counts were transformed to normality after excluding any extreme outliers. Fourteen Wnt genes showed detectable expression in our sample and were examined further for association with age using linear regression both individually and using stepwise selection models to identify independent signals. A Bonferroni-corrected alpha for the 14 tested genes (α=0.0036) was used. Six of the 14 tested genes showed significant univariate correlation with greater age [r (gene): 0.169 (APC), 0.179 (AXIN1), 0.222 (CTNNB1), 0.211 (GSK3b), -0.178 (LEF1), -0.166 (TCF1)]. When combined, expression of four genes was determined to be marginally (P<0.05) independently associated with age (AXIN1, CDH2, CTNNB1, TCF1). After correction for multiple testing, a 10-year greater age was independently associated with 4% greater CTNNB1 expression and 8% lower TCF1 expression respectively (both P<0.0001), and this association accelerated after age ≥60 years (p-interaction 0.01 and 0.07, respectively). Greater expression of CTNNB1 and lower expression of TCF1 may indicate decreased Wnt pathway signaling. These results are the first to suggest that aging may be associated with alterations in Wnt pathway signaling.

